# Editorial: Precision Medicine and Translational Research in Urological Oncology

**DOI:** 10.3389/fonc.2022.967278

**Published:** 2022-07-05

**Authors:** Rong Na, Jonathan Olivier, Gong-Hong Wei

**Affiliations:** ^1^ Division of Urology, Department of Surgery, Queen Mary Hospital, The University of Hong Kong, Hong Kong, Hong Kong SAR, China; ^2^ Department of Urology, Centre Hospitalier Universitaire de Lille (CHU) Lille, Claude Huriez Hospital, University of Lille, Lille, France; ^3^ Fudan University Shanghai Cancer Center, Department of Biochemistry and Molecular Biology, School of Basic Medical Sciences, Shanghai Medical College of Fudan University, Shanghai, China

**Keywords:** precision medicine, translational research, prostate cancer, bladder cancer, kidney cancer, genetics, omics

Precision medicine has been widely acknowledged by scientists and physicians nowadays. The application of high-throughput omics technologies has provided a panorama of diseases at multiple levels. Thus, many diseases are reclassified based on their molecular spectrums for the purposes of personalized management. In recent years, plenty of research has been conducted to translate the omics/basic studies to clinical practice and precision treatment. Machine learning/artificial intelligence has been widely applied in the field of biomedical studies which helps overcome the large amount of manual computation. These approaches encourage the development of useful and practical clinical tools for personalized disease management.

The current Research Topic includes 10 multidisciplinary original research focusing on the “Precision Medicine and Translational Research in Urological Oncology”. Each study has addressed some of the critical issues in the field or provided useful tools for potential clinical applications. To be more specific, this special issue includes: 1) studies involving the discovery, mechanisms, and applications of biomarkers for genitourinary cancer diagnosis or prognosis (Dong et al., Gu et al., Lin et al., Yang et al. and Jiang et al.); 2) development of clinical tools using omics-based, big data, or machine learning approaches (Li et al, Ning et al, Chen et al., Wang et al., and Zuo et al.).

A series of original research in this issue investigated the discovery, mechanisms, and applications of biomarkers for genitourinary cancer diagnosis or prognosis. Dong et al. presented a comprehensive study bridging the GWAS findings and biological mechanisms in prostate cancer. They reported an interesting dual-directional regulation of androgen signaling pathway by a non-coding risk-associated germline common variant at TERT promoter region. The variant, therefore, could also serve as a potential biomarker for predicting prostate cancer risk and outcomes. A phenome-wide exposed-omics analysis of the risk factors for prostate cancer with subsequent causal inference by Mendelian randomization from Gu et al. discovered four potential novel biomarkers for prostate cancer. A germline common variant in 8q24.21 was identified to be associated with [-2]proPSA by Lin et al. *via* a GWAS in a Chinese cohort. This explains the phenomenon that baseline level of [-2]proPSA and its derivative prostate health index (phi) varies largely in individuals. It may also help us establish a genetic-adjusted biomarker with additional translational study in the future. As a significant biomarker for cancer prognosis, circulating tumor cells (CTCs) has been widely applied in clinical practice. Yang et al. identified a subtype CTC called mesenchymal CTC that was significantly associated with poor survival of oligometastatic hormone-sensitive prostate cancer. Besides the studies in prostate cancer, Jiang et al. performed a systematic evaluation of a bladder cancer prognostic biomarker CX3CL1 including the functional study and the translational application.

Another series of important findings have been presented in this issue regarding the development of clinical tools using omics-based, big data, or machine learning approaches. A nomogram was established by Li et al. using clinical characteristics from big data for predicting the prognosis of renal cell carcinoma which was further confirmed in an independent cohort. At molecular level, a gene-based risk model based on large-scale omics studies was reported by Ning et al. which would have potential value for predicting the response to immune therapy in clear cell renal cell carcinoma. Meanwhile, Chen et al. provided an artificial intelligence approach to identify epithelial–mesenchymal transition subtype (poorer survival) of clear cell renal cell carcinoma *via* machine-based evaluation of H&E slides. Radiomics and imaging genomics is another popular topic during the recent years. In the current issue, Wang et al. reported an interesting imaging-based index named computed tomography (CT) fat attenuation index (FAI) for predicting renal cell carcinoma prognosis. This index was also found to be correlated with a molecular subtype of cancer. Incorporating with deep learning approach, Zuo et al. introduced a powerful tool distinguishing papillary renal cell carcinoma and chromophobe renal cell carcinoma based on CT imaging. These two subtypes of renal cell carcinoma are usually difficult to be distinguished manually *via* CT.

In summary, these 10 original studies collected in the current Research Topic provide invaluable evidence in terms of the precision medicine and translational research in different genitourinary cancers. Further translational studies are particularly promising based on the findings of these studies to apply the novel tools for personalized management to the diseases.

## Author Contributions

RN wrote the editorial. JO and GW reviewed and finalized the editorial. All authors contributed to the article and approved the final version.

## Conflict of Interest

The authors declare that the research was conducted in the absence of any commercial or financial relationships that could be construed as a potential conflict of interest.

## Publisher’s Note

All claims expressed in this article are solely those of the authors and do not necessarily represent those of their affiliated organizations, or those of the publisher, the editors and the reviewers. Any product that may be evaluated in this article, or claim that may be made by its manufacturer, is not guaranteed or endorsed by the publisher.

